# Adolescents’ Online Connections with Friends during COVID-19: An Assessment of the Forms of Communication and Their Association with Emotional Adjustment

**DOI:** 10.3390/children10081281

**Published:** 2023-07-26

**Authors:** Allie M. Spiekerman, Yue Guo, Jessica Payton, Nicole Campione-Barr, Sarah E. Killoren, Wendy M. Rote, Amanda J. Rose

**Affiliations:** 1 Department of Psychological Sciences, University of Missouri, Columbia, MO 65211, USA; aspiekerman@mail.missouri.edu (A.M.S.); ygz9q@mail.missouri.edu (Y.G.); jpayton@mail.missouri.edu (J.P.); campionebarrn@missouri.edu (N.C.-B.); killorens@missouri.edu (S.E.K.); 2 Department of Psychology, University of South Florida, Tampa, FL 33620, USA; wmrote@usf.edu

**Keywords:** COVID-19, depressive symptoms, loneliness, flourishing, social media

## Abstract

The COVID-19 pandemic and subsequent stay-at-home orders limited adolescents’ ability to connect with friends in person, leading adolescents to rely on digital forms of communication to interact with friends. The present study (*N* = 168 adolescents ages 11–20, 51.40% female) examined the types of digital communication adolescents used to connect with friends during the pandemic stay-at-home orders and how each form of digital communication related to adolescents’ emotional adjustment. The results showed texting to be the most common way adolescents connected with friends. Boys were more likely than girls to talk with friends through social gaming. Synchronous forms of communication (i.e., texting, video calls, and social gaming) were associated with reduced loneliness and depressive symptoms and higher flourishing. Connecting with friends by posting or responding on social media was not associated with adolescent well-being. These results suggest that forms of digital communication that allowed adolescents to talk with friends in real time were particularly important for adolescents’ emotional well-being during the COVID-19 pandemic.

## 1. Introduction

In March 2020, the World Health Organization declared the SARS-CoV-2 (COVID-19) virus as a global pandemic. This led to widespread lockdowns and stay-at-home orders that significantly disrupted adolescents’ lives. Across multiple countries, increases in mental health problems have been found among youth during these lockdowns [1,2,3,4], including increases in depressive symptoms [5,6,7] and loneliness [8,9], as well as lower flourishing (i.e., adolescents’ feelings of fulfillment and meaning) [10]. Increases in emotional adjustment problems were likely driven by the unique stressors during the pandemic, such as increased family conflict [11], increased COVID-related stress [12,13], and financial concerns [12]. 

One of the most salient stressors among youth during the pandemic was the disruption of in-person interactions with friends [12,13]. The disruption of adolescents’ interaction with friends was problematic given that friendship is an important type of relationship and friends provide sources of support during adolescence [14,15]. Indeed, experiences in friendships are consistently found to be related to adolescent depressive symptoms and loneliness [16] as well as positive indices of well-being, such as flourishing [17]. 

Importantly, one way that adolescents could still interact with friends during stay home orders was through digital communication. In general, adolescents’ use of technology for interacting with others during COVID-19 lockdowns and stay-at-home orders has been associated with better well-being [18]. However, less is known about the impact of adolescents’ digital interactions with friends specifically. 

The present study considered specific ways that adolescents connected with friends (e.g., texts, social media, and gaming) and had three goals: (1) identify which forms of digital communication adolescents most often used to connect with friends during shutdown orders; (2) test whether adolescents who did and did not use specific types of digital communication to connect with friends differed in terms of their emotional well-being (i.e., depressive symptoms, loneliness, and flourishing); and (3) examine the roles of gender and age differences in terms of the types of digital communication used to connect with friends and their associations with well-being. Data were collected in the United States, where widespread stay-at-home orders began in March 2020, which limited adolescents’ ability to connect with friends in person [19]. This context was appropriate for studying digital communication as digital communication is common among adolescents living in the United States, with over 89% of adolescents in the United States owning a smartphone [20].

### 1.1. Different Forms of Digital Communication with Friends during Pandemic Stay-at-Home Orders

During the COVID-19 stay-at-home orders, connecting with friends shifted from in-person communication to digital spaces, including text messaging, video calls, posting and responding on social media platforms, voice-only calls, and social gaming (i.e., playing video games with others using video chats or instant messaging while playing). Little research has addressed the types of communication that adolescents used most often to connect with friends often during the COVID-19 stay-at-home orders. However, the results from a few studies indicate that adolescents’ time spent on video calls, social media, voice calls, and social gaming with others increased in general [21]. In these studies, social media use and social gaming were most common, followed by texting and video calls [21,22,23]. The present study extends this body of research to focus on adolescents’ digital interactions specifically with *friends*. Although adolescents may have digitally communicated with friends during the pandemic using the same methods that they communicated with others, as indicated in the previous studies, this possibility had not been tested.

### 1.2. Different Forms of Digital Communication and Emotional Well-Being during Pandemic Stay-at-Home Orders

The associations between adolescents engaging in different forms of digital communication with friends and their emotional well-being were also tested. Two forms of problematic emotional adjustment, depressive symptoms and loneliness, were considered. We also assessed flourishing, which is defined as one’s levels of fulfillment and meaning in various aspects of their life, which encompasses adolescents’ overall feelings about their relationships, meaning, engagement, and accomplishments [24,25].

Two major categories can be used to classify different types of digital communication. Asynchronous communication, such as posting on social media and responding to others’ posts on social media, is communication put forth without expecting an immediate response [26]. Synchronous digital communication, however, involves communicating in real time, with friends responding immediately or within brief periods of time, and it includes video calls, voice-only calls, and social gaming [26]. Text messaging can be either asynchronous or synchronous, depending on whether responses are immediate or not. 

Pre-pandemic studies suggest that synchronous digital communication is more strongly related to positive emotional adjustment than asynchronous communication. For example, synchronous communication with friends, such as the forms listed previously, has been associated with positive mood [27], improved well-being [28], and lower depressive symptoms [29,30] among adolescents. In contrast, in terms of asynchronous communication, at least one study found that posting on social media was related to lower depressive symptoms through higher friend support [31]. Still, other forms of asynchronous social media use, such as browsing on social media, have not been consistently shown to be associated with positive well-being. Synchronous communication may be more strongly associated with emotional well-being because it involves a more engaged, reciprocal connection between friends than synchronous communication [32].

Emerging research suggests that connecting with others in general (not friends in particular) during the pandemic was associated with emotional adjustment. Some of these studies considered digital communication in general without specifying the type of digital communication. For example, one study found that digital communication involving one-on-one interactions buffered adolescents from loneliness during the pandemic [18]. Another study found that connecting with others online, and associated perceptions of support, were associated with reduced loneliness and depressive symptoms among adolescents [33]. 

Two other studies did consider the specific types of digital communication adolescents used. One study found that, in general, engaging in synchronous digital communication, namely texting and video calls, with others was associated with positive mood and lower depressive symptoms [34]. Another study found that self-disclosure to friends on social media was associated with higher flourishing [35]. The amount of time spent on social media, however, was not associated with depressive symptoms. 

In the present study, we assessed whether engaging in different types of digital communication with friends during the pandemic was associated with depressive symptoms, loneliness, and flourishing. We expected that communicating via video calls, voice-only calls, and social gaming would be associated with lower depression and loneliness and greater flourishing because these forms of communication are synchronous. In contrast, engaging in asynchronous forms of communication, namely posting on social media and responding to friends’ posts on social media, were not expected to be related to positive emotional adjustment. In addition, although texting can be synchronous or asynchronous, we expected that texting with friends would be associated with lower depression and loneliness and greater flourishing given the past study indicating that, in general, texting with others during the pandemic was associated with more positive emotional adjustment [34].

### 1.3. The Role of Gender and Age

Gender and age differences are consistently found in adolescents’ friendships. Girls are more likely to engage in intimate disclosure in their friendships, whereas boys tend to value companionship and engage in more shared activities, such as sports [36]. Additionally, friendship support and closeness tend to be higher among older adolescents than among younger adolescents [37]. 

Consistent with these findings, research prior to the pandemic indicated that girls texted and used social media more than boys [38,39], and boys engaged in more social gaming than girls [40]. During the pandemic, girls also were found to use social media more than boys, and boys were found to engage in social gaming more than girls [23]. We expected similar findings for connecting with friends through texting, using social media, and gaming. Gender differences were also tested for video calls and voice-only calls. 

In terms of age, some studies suggest that texting and social media use increase after early adolescence [41,42]. It was also speculated that, during the pandemic, older adolescents used texting and social media more than younger adolescents. Age differences in video calls, voice-only calls, and social gaming were also examined.

In addition, we investigated whether gender and age moderated the associations between adolescents using specific forms of digital communication with friends and their well-being. Because girls tend to be more sensitive to interpersonal experiences than boys [43], the associations between connecting with friends online and adjustment may be stronger for girls than boys. Similarly, because the importance of friends as a source of social support increases during adolescence [44], the associations may be stronger for older adolescents than their younger peers. Alternatively, because interacting with friends is a universal aspect of development. The emotional benefits of connecting with friends online may be similar across girls and boys and the age of adolescents. Accordingly, we had no firm hypotheses regarding gender and age as moderators.

### 1.4. Summary of Hypotheses

To summarize, we expected adolescents to report using a variety of methods of digital communication with friends during the pandemic, with the most common methods being texting and social media. In addition, we expected synchronous forms of communication, namely texting, video calls, voice-only calls, and social gaming, to be more strongly associated with emotional well-being than posting and responding on social media, which are asynchronous forms of communication. We also examined gender and age differences in engaging in specific forms of digital communication with friends and their role in the associations between using these forms of communication and emotional adjustment.

## 2. Materials and Methods

### 2.1. Participants

Participants were 168 adolescents (51.40% female) aged 11 to 20 years old (*M* = 16.21, *SD* = 2.03), and the cohort included 69 pairs of siblings. The majority identified as White and non-Hispanic (58.10%; 11.17% Black/African American, 7.82% Hispanic, 3.35% Asian, 3.35% American Indian/Alaska Native, 2.23% Hawaiian/Pacific Islander, and 20.67% Other or no response). The median household income was USD 70,000–99,999, and parents were overall highly educated (64.8% had a 4-year degree or higher).

### 2.2. Procedure

The participants in the current study were all participants in previous studies conducted in 2017–2018. Adolescents either participated in a previous data collection in Missouri (PIs, N. Campione-Barr and S. Killoren; for example, see [45]) or in southern Florida (PI, W. Rote; for example, see [46]). These adolescents and their families were contacted again in June/July 2020 and invited to participate in an online data collection focused on adolescent coping and close relationships during COVID-19. During this time, stay-at-home guidelines were in place at both data collection sites. Participants under 18 were required to have parental consent and provided their own consent if they were over 18. All participants completed online Qualtrics surveys and received Amazon gift cards as compensation. 

### 2.3. Measures

#### 2.3.1. Communication with Friends

Participants completed the “Adolescent Social Connection & Coping During COVID Questionnaire” Version 4/5/2020 [47]. One item was analyzed for the present study: “During April 2020 stay-at-home orders, which of the following methods did you use to connect with friends, without seeing them in person?” Response options included (1) messaging/texting, (2) video calls, (3) voice-only calls, (4) posting on social media, (5) liking or responding to posts friends made, (6) playing online games with them, and (7) other. The first six response options were coded 1 if selected and 0 if not selected, and the participants were allowed to select as many as applicable. 

#### 2.3.2. Depressive Symptoms

Participants completed the Center for Epidemiologic Studies Depression Scale (CES-D) [48], a twenty-item scale with items rated on a scale of 1 to 4 and four reverse-coded items. Each item asked adolescents to rate how often they experienced the symptom or feeling described in each item in the past week (e.g., “I felt depressed”; 1 = rarely or none of the time; 4 = most or all of the time). Scores were the mean of all items, with higher scores indicating greater depressive symptoms (α = 0.91). 

#### 2.3.3. Loneliness

Participants completed the UCLA Loneliness Scale Version 3 [49], a twenty-item scale with items rated on a scale of 1 to 4 and nine reverse-coded items (e.g., “I feel isolated from others;” 1 = never; 4 = often). Scores were the mean of all items, with higher scores indicating more loneliness (α = 0.94). 

#### 2.3.4. Flourishing

Participants completed the Flourishing Scale [50], an 8-item scale with items rated on a scale of 1 to 7. Each item included a statement and asked youths to rate the extent to which they agreed with it (e.g., “I lead a purposeful and meaningful life”; 1 = strongly disagree; 7 = strongly agree). Scores were the mean of all items, with higher scores indicating greater flourishing (α = 0.93). 

## 3. Results

### 3.1. Percentage of Adolescents Who Engaged in Each Type of Digital Communication

The total number of participants reporting using each form of digital communication to connect with friends is displayed in Table 1. Most participants reported texting to connect with friends (85.6% of the sample), and about half of the participants reported using video calls to connect with friends (52.4%). Approximately 30–40% of adolescents reported using voice-only calls (32.1%), posting on social media (38.1%), responding to friends’ social media posts (41.1%), and social gaming (36.3%). These descriptive results are presented in Table 1.

To examine whether there were gender and age differences in engaging in each form of communication, hierarchical logistic regressions were computed for each form of connecting with friends. In the analyses, the dependent variable was whether or not participants engaged in that form of communication. Gender and age were entered as main effects (Step 1), followed by the age X gender interaction (Step 2). The age X gender interaction was not significant for any models, so only the main effects are reported. All analyses were conducted in R 4.3.0.

Gender and/or age differences were found for video calls, posting and responding on social media, and social gaming. Younger adolescents were more likely than older adolescents to communicate with friends using video calls (*β* = −0.07, *t* = −3.90, *p* < 0.01, OR = 0.93), and girls were more likely than boys to have video calls with friends (*β* = 0.28, *t* = 3.84, *p* < 0.01, OR = 0.1.32). Girls reported posting on social media (*β* = 0.15, *t* = 2.05, *p* < 0.05, OR = 1.17) and responding to friends’ social media posts (*β* = 0.28, *t* = 3.75, *p* < 0.01, OR = 1.32) more than boys, and boys reported social gaming with friends more than girls (*β* = −0.45, *t* = −6.91, *p* < 0.001, OR = 0.64). No gender or age differences were found for the most common (texting) and least common (voice-only calls) forms of digital communication.

### 3.2. Forms of Digital Communication with Friends and Emotional Adjustment

To analyze the associations between each form of digital communication with friends and emotional adjustment, hierarchical regression models were established. Separate analyses were conducted for depressive symptoms, loneliness, and flourishing. In each model, gender and age were entered as main effects/control variables (Model 1; the results are presented in tables but not reported in text), followed by the main effect of the type of digital communication (Model 2), and then the interactions of gender and age with the form of Communication (Model 3). Age was mean-centered prior to being included in the interaction models. 

#### 3.2.1. Depressive Symptoms

Only one significant effect was found for depression. Having video calls with friends was associated with significantly lower depressive symptoms (*β* = −0.20, *t* = −2.48, *p* < 0.05). The associations with depression were not significant for texting, voice-only calls, posting on social media, responding to friends’ social media posts, or social gaming. No interactions were observed with gender or age. The full results regarding depressive symptoms are presented in Table 2.

#### 3.2.2. Loneliness

The results regarding loneliness are shown in Table 3. No significant effects were found for voice-only calls, posting on social media, or responding to friends’ social media posts. However, significant effects were found for video calls and social gaming. Connecting with friends through video calls (*β* = −8.72, *t* =−4.43, *p* < 0.001) and social gaming (*β* = −5.11, *t* = −2.26, *p* < 0.05) were both associated with lower loneliness. 

Although the main effect of texting was not significant, there was a significant age X texting interaction (*β* = 3.23, *t* = 2.08, *p* < 0.05). To interpret this interaction, we used the Johnson–Neyman regions of significance approach rather than the more common approach of testing simple slopes. Simple slope analyses provide information regarding whether the effects are significant at chosen levels of the moderator (e.g., one SD above and below the mean) but do not identify the specific value of the moderator at which the effect becomes significant. In contrast, the Johnson–Neyman approach allowed us to determine at which age texting became significantly associated with loneliness. This analysis, with whether or not adolescents used texting as the predictor and age as the moderator, revealed that this value was the age of 14, indicating that texting was only associated with lower loneliness for adolescents 14 years old and younger. 

#### 3.2.3. Flourishing

The results of flourishing analyses are presented in Table 4. For flourishing, significant effects were found for texting and video calls. Video calls with friends (*β* = 0.72, *t* = 3.79, *p* < 0.001) and texting with friends (*β* = 7.07, *t* = 2.72, *p* < 0.01) were associated with greater flourishing. In addition, for texting, the interaction with age was significant (*β* = −0.42, *t* = −2.58, *p* < 0.05). The Johnson–Neyman regions of significance approach was used to probe the interaction. For this analysis, whether or not the adolescent reported using texting to connect with friends was the predictor, and age was the moderator. This test revealed that texting was associated with greater flourishing for adolescents who were 16 and younger but not for older adolescents. No significant effects of flourishing were observed for voice-only calls, posting on social media, responding to posts on social media, or social gaming.

## 4. Discussion

The current study examined the ways adolescents used technology to connect with friends during COVID-19 stay-at-home orders and how these methods related to adolescent emotional adjustment. COVID-19 led to lockdowns and stay-at-home orders that limited adolescents’ abilities to connect with friends in person. The pandemic was also associated with a rise in depressive symptoms, loneliness, and lower flourishing among youth [5,6,9,10]. Although several studies have examined adolescents’ use of digital communication and technology during COVID-19 more generally [18,21,23], little is known about the ways youths connected with friends during COVID-19 stay-at-home orders and how these methods related to their emotional adjustment. We examined six forms of digital communication, four of which involved synchronous communication (video calls, voice-only calls, and social gaming), and two involved asynchronous communication (posting on social media and responding to friends’ social media posts). There is some ambiguity regarding whether texting is synchronous or asynchronous. Additionally, we focused on three separate emotional adjustment outcomes: depressive symptoms, loneliness, and flourishing.

In terms of the methods of digital communication adolescents used to connect with friends during the pandemic, we found that text messaging was the most common method, and voice-only calls were the least common method. Except for video calls, which younger adolescents were more likely to use than older adolescents, none of the types of communication to connect with friends varied by age. Older adolescents were expected to use most forms of digital communication at greater rates than younger adolescents because the salience of friends as a source of support tends to increase throughout adolescence [44]. Additionally, older adolescents may have more access to digital devices. However, these effects were not observed. Perhaps older adolescents were able to see friends in person more than younger adolescents and did not need to use as many digital forms of communication to connect with friends. Additionally, parents may have been less stringent on younger adolescents’ device use compared with before the pandemic [51], thus allowing younger adolescents to use digital forms of communication at similar rates as older adolescents.

The effects of gender were consistent with the hypotheses. Girls were more likely to use social media to connect with friends (both posting and responding to friends’ posts), whereas boys were more likely to use social gaming to connect with friends. These findings are in agreement with previous research testing gender differences before the pandemic [38,40]. Interestingly, the rates of using text messaging and voice-only calls to connect with friends were similar among boys and girls. These forms of communication were the most and least common, respectively. Perhaps gender differences did not emerge because texting was so ubiquitous, whereas voice-only calls were so rare.

Also consistent with our expectations, significant associations were found between engaging in specific types of communication with friends and emotional adjustment. Specifically, significant effects were observed for two of the three types of synchronous communication. Video chatting with friends during stay-at-home orders was significantly associated with lower depressive symptoms, lower loneliness, and higher flourishing. Additionally, social gaming with friends was associated with lower levels of loneliness. Although voice-only calls also involve synchronous communication, they were not associated with emotional adjustment, perhaps because they were relatively rare. Overall, the findings are in line with research on synchronous communication before the pandemic [32] and are consistent with the possibility that the reciprocal nature of synchronous communication allows adolescents to feel more connected with friends.

As noted, texting is ambiguous in terms of whether it is synchronous or asynchronous. Interestingly, using text messaging to connect with friends was related to lower loneliness and higher flourishing but only among younger adolescents. This finding conflicts with the possibility that the effects would be especially strong for older adolescents given the increased salience of friends with age. Perhaps older adolescents were more likely to leave their houses during the pandemic than younger adolescents because they were more likely to have essential jobs (e.g., working in grocery stores, daycares) and more autonomy from parents and used this greater freedom to see friends in person. If this is the case, this could help explain why texting, which was the most common form of digital communication with friends, was especially closely linked with emotional adjustment for younger adolescents.

In contrast to synchronous forms of digital communication, connecting with friends via the two asynchronous forms of communication assessed, posting and responding on social media, was not associated with emotional well-being. Although these results support the hypothesis that asynchronous communication with friends would not be associated with positive emotional adjustment as strongly as synchronous forms of communication, the findings conflict with some previous research suggesting that using social media may have negative implications for adolescents’ mental health [52]. Perhaps the benefits of connecting with friends through social media during stay-at-home orders when in-person interaction with friends was limited outweighed the potential negative effects of social media use. 

Apart from the interactions found for texting, the associations with emotional adjustment did not vary by age or gender. Although differential associations may have been expected given girls’ greater emotional reactivity to social interactions [43], and the greater centrality of friends for older adolescents compared with their younger peers, our findings are in agreement with the previous literature suggesting that the associations between friendship experiences and emotional adjustment tend to hold across genders and ages [16]. Thus, our findings speak to the broad significance of synchronous communication with friends among adolescents during the pandemic.

### Limitations and Future Directions

The present study did have limitations. First, our measure for forms of digital communication with friends asked the participants to indicate whether or not they used each form of communication and did not ask them to elaborate on the amount of time spent using each method. Some research has found a curvilinear relationship between the time spent online and adolescent adjustment, with those who spend a little bit of time faring better than both those who use it excessively and those who do not use it at all [38]. Having these data would have been helpful for clarifying the associations of connecting with friends via different types of digital communication and emotional adjustment. 

Similarly, the measure used in this study did not allow for an examination of the specific nature of friends’ communication. One possibility is that synchronous forms of communication were associated with well-being because these methods allow for more self-disclosure and opportunities to provide social support [32]. Perhaps even more significant effects would have emerged had the extent of social support received from friends through each form of digital communication been assessed.

The findings also were not longitudinal. Although the research was motivated by the idea that connecting with friends would lead to positive changes in emotional adjustment, the direction of the effects cannot be known from this study. An alternative possibility is that youth who were already well adjusted were more likely to connect with friends using synchronous forms of digital communication. Although we cannot conclude that connecting with friends via synchronous online communication buffered adolescents from the negative mental health impacts of COVID-19, the data did allow for the identification of the types of digital communication that adolescents used with friends that were linked with positive emotional adjustment. 

In addition, although a detailed focus on social media was a strength of the study, we acknowledge that the associations between digital communication with friends and adolescent adjustment were not considered in the broader context of the pandemic. During the pandemic, many adolescents experienced a range of significant stressors that were associated with their emotional adjustment. Indeed, adolescents’ experiences of lifestyle changes, conflict with parents, and perceived stress have been found to be associated with emotional adjustment problems [11,53,54].

Finally, it is important to acknowledge the homogeneity of the sample in the current study. Participants were predominantly White, middle-class adolescents born and living in the United States. This sample facilitated the study of social media given that the vast majority of adolescents in this demographic group have access to smartphones and computers, and social media use is pervasive [20]. However, the results do not provide information about adolescents in the United States and elsewhere who do not have access to digital communication or adolescents living in parts of the world that did not impose stay-at-home guidelines. 

## 5. Conclusions

The present study reveals that using technology to connect with friends during the pandemic is associated with positive emotional adjustment among adolescents. These findings highlight that technology use in adolescence is not inherently negative but rather depends on the context of its use. Moreover, in the present study, we found that using technology to connect with friends was positively associated with well-being *only* if communication was synchronous. Even beyond the pandemic, the findings suggest that parents should endorse technology use that involves a reciprocal and engaged connection with friends more than technology involving asynchronous communication with friends. Continued research should focus on the specific ways connecting with friends via synchronous forms of digital communication can help promote positive emotional adjustment among adolescents. 

## Figures and Tables

**Table 1:** Frequencies of adolescents reporting using each form of digital communication.

Form of Communication	Boys	Girls	Total	Mean Age
Texting				
Yes	70 (85.4%)	24 (89.5%)	147 (87.5%)	16.29
No	12 (14.6%)	58 (10.5%)	21 (12.5%)	15.67
Voice-Only Calls				
Yes	24 (29.3%)	30 (34.9%)	54 (32.1%)	16.37
No	58 (70.7%)	56 (65.1%)	114 (67.9%)	16.13
Video Calls				
Yes	32 (39%)	56 (65.1%)	88 (52.4%)	15.72
No	50 (61%)	30 (34.9%)	80 (47.6%)	16.75
Post on Social Media				
Yes	25 (30.5%)	39 (45.3%)	64 (38.1%)	16.03
No	57 (69.5%)	47 (54.7%)	104 (61.9%)	16.31
Respond to Friends’ Social Media Posts				
Yes	22 (26.8%)	47 (54.7%)	69 (41.1%)	16.36
No	60 (73.2%)	39 (45.3%)	99 (58.9%)	16.10
Social Gaming				
Yes	49 (59.8%)	12 (14%)	61 (36.3%)	15.89
No	33 (40.2%)	74 (86%)	107 (63.7%)	16.39

**Table 2:** Regression results regarding depressive symptoms.

	Texting		Voice-Only Calls		Video Calls		Post on Social Media		Respond to Social Media Posts		Social Gaming	
	*Β*	*t*-Value	*β*	*t*-Value	*Β*	*t*-Value	*Β*	*t*-Value	*β*	*t*-Value	*β*	*t*-Value
Model 1												
Gender	0.41	5.61 ***	0.41	5.61 ***	0.41	5.61 ***	0.41	5.61 ***	0.41	5.61 ***	0.41	5.61 ***
Age	0.01	0.72	0.01	0.72	0.01	0.72	0.01	0.72	0.01	0.72	0.01	0.72
Model 2												
Form of Communication	0.02	0.14	0.03	0.32	0.20	2.48 *	−0.04	−0.57	0.05	0.67	−0.04	−0.42
Model 3												
Gender X Communication	0.30	1.33	0.20	1.22	0.12	0.76	0.17	0.27	0.19	10.17	0.04	0.19
Age X Communication	0.10	1.73 ^†^	0.03	0.76	0.01	0.19	0.02	0.62	−0.01	−0.15	−0.07	−1.65

Note: Gender was coded as 0 = boys and 1 = girls. Communication was coded as 0 = did use this form of communication to connect with friends and 1 = did not use this form of communication to connect with friends. ^†^ *p* < 0.1, * *p* < 0.05, and *** *p* < 0.001.

**Table 3:** Regression results regarding loneliness.

	Texting		Voice-Only Calls		Video Calls		Post on Social Media		Respond to Social Media Posts		Social Gaming	
	*Β*	*t*-Value	*β*	*t*-Value	*Β*	*t*-Value	*Β*	*t*-Value	*β*	*t*-Value	*Β*	*t*-Value
Model 1												
Gender	7.78	4.05 **	7.78	4.05 ***	7.78	4.05 ***	7.78	4.05 ***	7.78	4.05 ***	7.78	4.05 ***
Age	0.54	0.49	0.54	0.49	0.54	0.49	0.54	0.49	0.54	0.49	0.54	0.49
Model 2												
Form of Communication	2.75	0.93	3.85	1.89 ^†^	8.72	4.43 ***	−0.59	−0.30	0.74	0.36	5.11	2.26 *
Model 3												
Gender X Communication	−10.16	1.74 ^†^	2.95	0.72	0.36	0.10	−2.06	−0.51	0.11	0.03	−1.54	−0.32
Age X Communication	−3.23	2.08 *	0.46	0.43	0.24	0.25	0.58	0.56	−0.83	−0.77	−0.35	−0.33

Note: Gender was coded as 0 = boys and 1 = girls. Communication was coded as 0 = did use this form of communication to connect with friends and 1 = did not use this form of communication. ^†^ *p* < 0.1, * *p* < 0.05, ** *p* < 0.01, and *** *p* < 0.001.

**Table 4:** Regression results of flourishing.

	Texting		Voice-Only Calls		Video Calls		Post on Social Media		Respond to Social Media Posts		Social Gaming	
	*β*	*t*-Value	*β*	*t*-Value	*β*	*t*-Value	*β*	*t*-Value	*β*	*t*-Value	*β*	*t*-Value
Model 1												
Gender	−0.46	−2.53 *	−0.46	−2.53 *	−0.46	−2.53 *	−0.46	−2.53 *	−0.46	−2.53 *	−0.46	−2.53 *
Age	0.06	1.25	0.06	1.25	0.06	1.25	0.06	1.25	0.06	1.25	0.06	1.25
Model 2												
Form of Communication	−0.51	1.77 ^†^	−0.10	−0.49	−0.72	−3.79 ***	−0.13	−0.69	−0.25	−1.26	0.17	0.79
Model 3												
Gender X Communication	0.31	0.55	−0.28	−0.71	0.05	0.13	0.28	0.71	0.02	0.05	0.25	0.54
Age X Communication	−0.42	−2.58 *	0.12	1.11	−0.02	−0.18	−0.13	−1.35	0.04	0.44	0.15	1.44

Note: Gender was coded as 0 = boys and 1 = girls. Communication was coded as 0 = did use this form of communication to connect with friends and 1 = did not use this form of communication. ^†^ *p* < 0.1, * *p* < 0.05, *** *p* < 0.001.

## Data Availability

Due to privacy concerns, data and analysis codes are available upon request.
